# Antibody responses to feline leukaemia vaccination: exploring the effect of sex, boosting schedule, site of administration and vaccine type

**DOI:** 10.1177/1098612X251353080

**Published:** 2025-09-04

**Authors:** Mark E Westman, Yasmin Parr, Caitlin Martin, Eliza Wuestner, Stefanie Pan, Jacqueline M Norris, Mike McDonald, Dawn Dunbar, William Weir, Evelyn Hall, Mizuho Nakamura, Nerida Atkin, Rabia Hajjar, Maira Nascimento Meggiolaro, David Hughes, Richard Malik, Margaret J Hosie

**Affiliations:** 1 Sydney School of Veterinary Science, The University of Sydney, Sydney, NSW, Australia; 2 Sydney Institute for Infectious Diseases, The University of Sydney, Sydney, NSW, Australia; 3 MRC, Centre for Virus Research, University of Glasgow, Glasgow, Scotland, UK; 4 Veterinary Diagnostic Services, School of Biodiversity, One Health and Veterinary Medicine, University of Glasgow, Glasgow, Scotland, UK; 5 Cat Protection Society of NSW, Newtown, NSW, Australia; 6 Concord Veterinary Hospital, Sydney, NSW, Australia; 7 Centre for Veterinary Education, The University of Sydney, Sydney, NSW, Australia

**Keywords:** Antibodies, feline leukaemia virus, fibrosarcoma, field study, humoral immune response, injection-site sarcoma, retroviral infection, vaccination, veterinary science

## Abstract

**Objectives:**

Historically, vaccines have been administered in the dorsal interscapular region of cats (the ‘scruff’ of the neck) owing to easy access to the subcutaneous space. In response to concerns about sarcomas developing at injection sites (feline injection site sarcomas [FISSs]), and a possible association between feline leukaemia virus (FeLV) vaccination and the development of FISS, alternative FeLV vaccination sites such as the distal left hindlimb and tail have been proposed by influential vaccination bodies and various key opinion leaders. There is a dearth of evidence, however, to demonstrate the development of a comparable immune response after FeLV vaccination in these sites.

**Methods:**

This field study was undertaken to investigate the FeLV anti-surface unit (SU) antibody response in FeLV-uninfected cats inoculated with one of three different FeLV vaccines (Fel-O-Vax 5, Fel-O-Vax Lv-K or Leucogen FeLV), administered in one of three different anatomical locations (‘scruff’, left distal hindlimb or tail). Kittens were sampled at three different time points, 1 month apart (T0, T1, T2) and again 12 months later (T12). Testing with a published anti-SU ELISA to detect FeLV-A and FeLV-B antibody responses to vaccination was performed. Antigen p27 testing, PCR testing to detect FeLV proviral DNA and neutralising antibody (NAb) testing to identify any FeLV-infected or FeLV-exposed animals were also performed.

**Results:**

A total of 125 kittens were recruited and allocated into one of nine vaccine groups, with 105 kittens completing the initial course of vaccinations and blood draws, and 83 cats returning for T12 sampling. No progressive or regressive FeLV infections were detected in the entire kitten or adult cohorts. A total of 14 (11%) kittens and two (2%) adults tested FeLV NAb-positive. Females had higher (approximately 1.6-fold) post-vaccinal FeLV-A and FeLV-B antibody concentrations compared with males (*P* = 0.003 and 0.009, respectively). An anamnestic response (‘booster’ effect) was observed, with FeLV-A and FeLV-B antibody levels higher at T2 (day 56) after two primary vaccine doses than at T1 (day 28) after one dose (*P* = 0.004 and *P* <0.001, respectively). No biologically significant differences in FeLV antibody concentrations were found between the different sites of vaccination or vaccine formulations. Tail injections produced fewer vaccine ‘non-responders’ against FeLV-A at T2 than scruff and hindlimb vaccination (*P* = 0.020), possibly because tail injections were actually intramuscular, due to a lack of subcutaneous space in the tail.

**Conclusions and relevance:**

FeLV vaccines can be administered in the scruff, left hindlimb or tail of cats, with comparable antibody responses observed across all sites. This result will assist veterinarians in making evidence-based recommendations about possible sites for FeLV vaccinations.

## Introduction

Feline leukaemia virus (FeLV) is a *Gammaretrovirus* with global distribution.^1^ Cats exposed to FeLV experience one of three main clinical outcomes: (1) abortive infection, (2) regressive infection or (3) progressive infection. An abortive FeLV infection is characterised by complete clearance of the virus before proviral integration occurs.^2^ Regressive FeLV infection occurs after a partially effective immune response, when an initial transient viraemia is cleared, but not before provirus has integrated into the cat’s DNA, where it persists for life.^3^ Progressive infections are characterised by persistent viraemia, ongoing high levels of proviral DNA and viral RNA, with an associated poor prognosis.^1^ It is generally accepted that most progressively infected cats succumb to aplastic anaemia, lymphoma, leukaemia or other myeloproliferative diseases within 3 years of infection.^1,4^ Recently, however, substantially longer survival has been demonstrated in some progressively infected cats with lower viral burdens (also known as ‘low positives’).^5^

The prevalence of progressive FeLV infection has declined in many developed countries over the past few decades, considered attributable to regular rapid point-of-care (PoC) testing for FeLV infection and the isolation or euthanasia of FeLV-infected cats.^6–9^ In many other countries, however, rates of progressive infection have remained stable and high for decades.^6,10,11^ In Australia, the estimated prevalence of progressive infection has hovered at approximately 1–2% for almost 30 years.^12,13^ A recent study, which included FeLV neutralising antibody (NAb) and proviral DNA PCR testing in addition to antigen testing, reported that 13% of pet cats in Australia with outdoor access were infected with or had been exposed to FeLV.^14^ Maintenance of FeLV infection in client-owned populations is likely to be, in part, due to higher rates of infection in unowned and feral cats, creating potential FeLV ‘hot spots’.^14,15^ Consequently, despite low prevalence rates in many countries, major vaccination guidelines continue to recommend FeLV as a core vaccine component for (1) young cats (aged <1 year) and (2) adult cats with outdoor access or exposure to cats with outdoor access.^16,17^

Historically, the most common injection site in cats has been the dorsal interscapular subcutaneous space (the ‘scruff’), as a cat may be restrained (with one hand) and injected simultaneously (using the other hand) with ease and safety. Vaccine licensing studies are typically based on vaccine administration into this subcutaneous space.^18,19^ In a practice setting, it is assumed that most of these injections are administered subcutaneously, although, in some cases, it is possible that part of the inoculum may be deposited deeper intramuscularly.

Feline injection-site sarcomas (FISSs) were first reported in the USA in 1991. At that time, lesions were typically located in the interscapular region,^20^ which provided circumstantial evidence that the sarcomas were linked causally to injections given at this location. Since then, incidence rates of one case per 1000–10,000 cats vaccinated (USA) and one case per 5000–12,500 vaccination visits (UK) have been suggested.^21–24^ Although data are hard to come by, we have the strong impression that the incidence of FISS is far lower now than it was 20 years ago. The incidence of FISS has not yet been reported for Australia, although sporadic cases of post-vaccinal FISS certainly occur.^25^ Adjuvanted (inactivated) vaccines, particularly FeLV and rabies vaccines containing aluminium, have been implicated in some studies as having potentially a greater risk of stimulating FISS formation than non-adjuvanted vaccines.^20,21,26–29^ Confusingly, other studies have not identified adjuvanted FeLV and rabies vaccines to be risk factors for FISS.^30,31^ The current American Animal Hospital Association (AAHA) and American Association of Feline Practitioner (AAFP; now the Feline Veterinary Medical Association, FelineVMA) guidelines for feline vaccination recommend subcutaneous injection of FeLV and rabies vaccines into the left distal hindlimb or distal tail to allow for amputation should post-vaccinal complications occur.^16^ One would imagine this would make recording the incidence of FISS in such locations simple, yet such data on distally located FISS are conspicuously lacking.

Despite the AAHA and AAFP’s recommendation to administer FeLV and rabies vaccines away from the usual site of injection (ie, the scruff), little work has been done to demonstrate a comparable immunological response to vaccination in these alternative sites. One pilot study, in which a modified-live virus (MLV; also known as attenuated) core vaccine containing feline panleukopenia virus (FPV) antigen and an inactivated rabies vaccine were administered either in the lateral hindlimbs below the stifles or the distal third of the tail, demonstrated no difference in antibody levels 1–2 months after vaccination. Both vaccination sites were well tolerated by cats, with minimal restraint required.^18^ Injection of a polyvalent live attenuated vaccine in the Houhai acupoint (the centre part of depression on the midline between the anus and the ventral base of the tail) induced the highest antibody responses in both rats and dogs compared with under the jaw, the popliteal fossa and the dorsal back, demonstrating a site-dependent difference in serological response to vaccination.^32^ In a retrospective analysis, we observed a significant difference in anti-surface unit (SU) FeLV antibody concentration in FeLV-unexposed cats vaccinated with Fel-O-Vax 5 (a polyvalent vaccine containing inactivated FeLV) vs Fel-O-Vax Lv-K (a monovalent FeLV vaccine), which may have been site-dependent.^33^ To date, there have been no prospective studies comparing the antibody response in cats vaccinated against FeLV in different anatomical sites. In contrast, this is an active research topic in human vaccinology.^34,35^

The aim of this study, therefore, was to determine the anti-FeLV serological response of kittens to a primary vaccination series with one of three commercially available FeLV vaccine formulations (Fel-O-Vax 5, Fel-O-Vax Lv-K and Leucogen FeLV) administered at one of three different anatomical sites, namely the scruff, distal hindlimb and tail.

## Materials and methods

### Study population

Healthy mixed-breed kittens aged 8 weeks or older with no known history of exposure to FeLV were recruited from a rescue organisation in Sydney, New South Wales (NSW) between January and August 2020, while awaiting rehoming. The aim was to recruit 135 kittens in total, randomly allocating them into 1/9 groups of 15 animals each. Each group was defined by having a different site of vaccination, either (1) the scruff, (2) left hindlimb or (3) tail, and vaccine formulation, either (1) Fel-O-Vax 5, (2) Fel-O-Vax Lv-K or (3) Leucogen FeLV (Table 1). Kittens were enrolled into groups 1–9 consecutively, in order of presentation (ie, kitten 1 = group 1, kitten 2 = group 2 and so on; group allocation restarted after nine kittens).

**Table 1:** Study groups and proposed group sizes (n = 15 cats per group) (see the ‘Vaccination schedule and vaccines administered’ section for vaccine formulation and manufacturer details)

Vaccination site	Fel-O-Vax Lv-K* (n = 45)	Leucogen FeLV* (n = 45)	Fel-O-Vax 5 (n = 45)
Tail (n = 45)	Group 1 (n = 15)	Group 2 (n = 15)	Group 3 (n = 15)
Left hindlimb (n = 45)	Group 4 (n = 15)	Group 5 (n = 15)	Group 6 (n = 15)
Scruff (n = 45)	Group 7 (n = 15)	Group 8 (n = 15)	Group 9 (n = 15)

* Animals in these groups were concurrently administered Feligen 3 in the scruff, a modified-live virus core vaccine containing feline panleukopenia virus, feline herpesvirus-1 and feline calicivirus, in addition to the feline leukaemia virus (FeLV) vaccine administered into one of three different anatomical areas. Animals in groups 7 and 8, therefore, received two separate injections into the scruff

**Figure 1:**
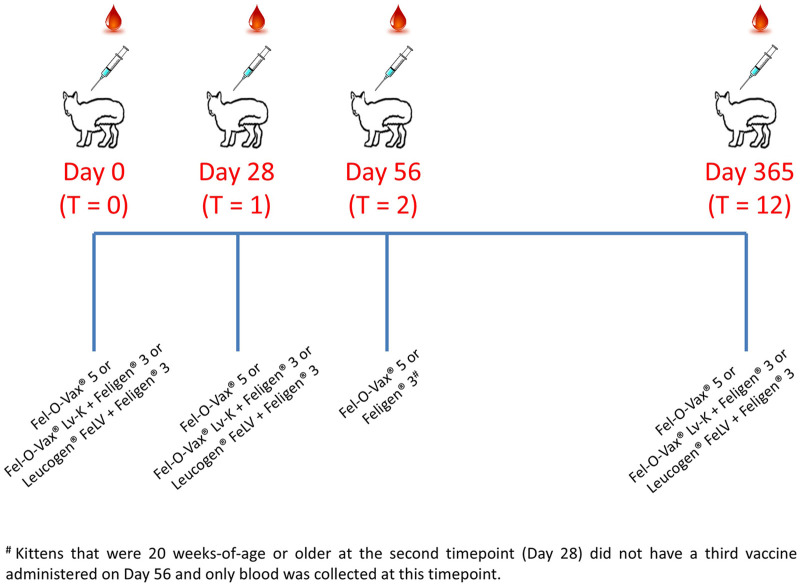
Vaccination and sampling schedule. Fel-O-Vax 5 is a polyvalent vaccine containing inactivated whole-feline leukaemia virus (FeLV) (and feline panleukopenia virus (FPV), feline herpesvirus-1 (FHV-1), feline calicivirus (FCV) and *Chlamydia felis*); Fel-O-Vax Lv-K is an inactivated monovalent whole-FeLV vaccine; Leucogen FeLV is an adjuvanted recombinant subunit p45 FeLV vaccine; and Feligen 3 is a modified-live virus vaccine containing FPV, FHV-1 and FCV antigens. See the ‘Vaccination schedule and vaccines administered’ section for further vaccine formulation and manufacturer details. T0 = day 0; T1 = day 28; T2 = day 56; T12 = day 365

Blood sampling was performed on day 0 (ie, the day of initial vaccination), day 28 and day 56, and 1 year later (day 365) (Figure 1). The vaccination schedule is detailed in the ‘Vaccination schedule and vaccines administered’ section. When cats were rehomed, owners were asked to decide whether to consent to ongoing participation in the study or to opt out. Owners who chose to participate agreed to confine their kittens indoors (with no outdoor access at all) for the duration of the primary vaccination course and committed to return for re-vaccination and sampling as per the study schedule.

All kittens were neutered before rehoming between day 0 (T0) and day 56 (T2).

### Vaccination schedule and vaccines administered

Animals enrolled in the study followed the vaccination and sampling schedule in Figure 1. For animals in the Fel-O-Vax 5 groups, three doses of vaccine were administered (days 0, 28 and 56). For the other groups, Fel-O-Vax Lv-K/Feligen 3 or Leucogen FeLV/Feligen 3 was administered on days 0 and 28, while on day 56 kittens were administered Feligen 3 only (ie, no FeLV vaccine). Therefore, adult cats in the study that had been administered Fel-O-Vax 5 as kittens received an additional vaccine booster against FeLV in contrast to the other vaccine groups. Kittens that were aged 20 weeks or older at the second vaccination (day 28) had blood collected but did not have a third vaccine administered on day 56, in accordance with vaccination recommendations.^16,17^

Fel-O-Vax 5 (Boehringer Ingelheim Animal Health Australia) is an adjuvanted polyvalent vaccine that includes an inactivated whole-virus (IWV) FeLV component. In addition to FeLV, it includes FPV, feline herpesvirus-1 (FHV-1), feline calicivirus (FCV) and *Chlamydia felis* inactivated antigens. The same vaccine is produced as Fel-O-Vax Lv-K IV in North America (Elanco Animal Health). Fel-O-VaxLv-K (Boehringer Ingelheim Animal Health Australia) is an adjuvanted monovalent IWV FeLV vaccine.

The FeLV component of both Fel-O-Vax 5 and Fel-O-Vax Lv-K was produced as an IWV vaccine from a single subtype isolate (FeLV-A/61E), by the same manufacturer in the same facility. The antigen potency specifications (minimum and maximum release titres) for FeLV were the same for both Fel-O-Vax 5 and Fel-O-Vax Lv-K.^33^

Leucogen FeLV (Virbac Animal Health) is a recombinant subunit p45 FeLV vaccine (p45 being the non-glycosylated form of gp70, the FeLV surface envelope glycoprotein), adjuvanted with aluminium hydroxide and saponin.^36^ The p45 antigen used was derived from the envelope glycoprotein of FeLV subgroup A.^37^

Animals vaccinated with Fel-O-Vax Lv-K and Leucogen FeLV were concurrently administered Feligen 3 (Virbac Animal Health), a core MLV (attenuated) vaccine containing FPV, FHV-1 and FCV into the scruff, irrespective of the FeLV vaccination site they were assigned in the study.

It was intended that vaccines administered into the left hindlimb were injected distal to the stifle. This may not have always been achieved, and some vaccinations may have been administered at the level of the stifle. All tail vaccinations were administered into the proximal third of the tail.

### Sample collection and processing

Approximately 1 ml of blood was collected from each kitten at three different time points (T0 = day 0, T1 = day 28 and T2 = day 56), with a final collection 12 months later (T12 = day 365) (Figure 1). Blood was collected from the jugular vein after application of a local anaesthetic cream, as required. Blood was aliquoted into two tubes, 0.5 ml into a lithium heparin (LH) tube and 0.5 ml into an EDTA tube. LH blood samples were centrifuged at 12,000 *g* for 3 mins, plasma transferred to plain cryovials and stored at –80°C. At the end of the study, frozen plasma samples were shipped on dry ice to Veterinary Diagnostic Services (VDS), University of Glasgow, for batch serology testing. Fresh EDTA-anticoagulated whole blood was stored at 4°C and used for PoC testing within 24–48 h of collection, with the remainder being stored as whole blood at –80°C for batch PCR testing at Veterinary Pathology Diagnostic Services (VPDS), the University of Sydney, at the end of the study.

### FeLV p27 antigen, FeLV proviral DNA and FeLV neutralising antibody testing for study inclusion/exclusion

Fresh EDTA-anticoagulated whole blood was used for testing with Anigen Rapid FIV/FeLV PoC test kits (BioNote) to simultaneously detect FeLV capsid protein (p27) and antibodies to feline immunodeficiency virus (FIV) envelope glycoprotein (gp40). In tests conducted on samples from Australian cats, Anigen Rapid FeLV has a reported specificity and sensitivity of 98% and 91%, respectively, and Anigen Rapid FIV a reported specificity and sensitivity of 100% and 100%, respectively.^38,39^

Semi-quantitative FeLV PCR (qPCR) testing was performed by VPDS to detect FeLV proviral DNA in leukocytes, as previously described.^40,41^ DNA was extracted from 200 µl of thawed EDTA-anticoagulated whole blood using a commercially available kit, the MagMAX CORE Nucleic Acid Purification Kit (Thermo Fisher Scientific), and 2 µl of extracted DNA (approximately 100 ng) was included in each 20 µl qPCR reaction. Samples with a cycle threshold (C_*T*_) value of less than 40 were considered qPCR-positive.

Virus neutralisation assays (VNAs) were performed by VDS to measure the presence of FeLV NAbs as previously described.^14,33,42^ Two-fold serial dilutions (1:4, 1:8, 1:16 and 1:32) of thawed LH plasma samples were tested. NAb titres were recorded as the reciprocals of the plasma dilution that reduced the count of FeLV focus-forming units (ffu) by 75% compared with the virus control incubated without plasma. A sample that returned a positive result at any dilution was considered NAb-positive. A reciprocal NAb titre of 4 was considered weakly NAb-positive, and a reciprocal titre of 32 was considered strongly NAb-positive.^14^ Reciprocal NAb titre results are reported herein. FeLV-vaccinated cats are not expected to produce NAb without prior natural or experimental exposure, irrespective of the type of FeLV vaccine administered.^4,14,33,42–47^

Samples collected at each of the four time points were tested using FeLV PoC, proviral qPCR and VNA to identify FeLV-infected (progressive and regressive infections) and FeLV-exposed animals (abortive infections). Progressive infections were defined as p27 antigen-positive and provirus-positive, regressive infections as p27-negative and provirus-positive, abortive infections as p27-negative, provirus-negative and NAb-positive, while p27-negative, provirus-negative and NAb-negative cats were considered FeLV-unexposed (Table 2).^14^ The possibility of NAb-positive results in kittens due to maternally derived antibodies (MDAs) instead of abortive infections could not be discounted. FIV-positive animals were retained in the study.

**Table 2:** Categories of feline leukaemia virus infection used in the study, according to test results

Infection category	p27 antigen	Proviral DNA	Neutralising antibodies	Interpretation
Progressive	+	+	+/−	Ongoing infection
Regressive	−	+	+/−	Controlled infection
Abortive	−	−	+	Cleared infection
Unexposed	−	−	−	No evidence of exposure

### FeLV serology testing for evaluation of antibody response after vaccination

Anti-SU antibody ELISA testing was performed at the Centre for Virus Research, University of Glasgow, to assess humoral response to FeLV vaccination, as previously described.^33,48^ Thawed LH plasma samples were tested for anti-SU antibodies, using both Fc-tagged FeLV-A SU and Fc-tagged FeLV-B SU as capture antigens. This was to ensure a comprehensive analysis of antibody responses against FeLV SU. The FeLV-B SU antibody response was interpreted as an antibody response to an epitope shared between FeLV-A and FeLV-B SU. Normalised optical density (NOD) values were determined using the formula:

$$NOD=\frac{\left(Sample\;OD-Negative\;control\;OD\right)}{\left(Positive\;control\;OD-Negative\;control\;OD\right)}$$

Samples were tested in triplicate and tests were repeated if the standard deviation for the three replicates was greater than 0.1. Anti-SU ELISA results were not classified as positive or negative; instead, the range of antibody responses against SU were compared among the cats tested. The assay positive control was a pooled sample of plasma from specific pathogen-free (SPF) cats that had been experimentally infected with FeLV, had developed a strong NAb response and tested persistently p27 antigen-negative, demonstrating recovery from infection. The negative control was a pooled sample of plasma from SPF cats that had negligible reactivity to FeLV SU by immunoblot.^33,48^

### Statistical analysis

Numerical analyses were performed using the statistical software Genstat 18th edition (VSN International) and R Version 4.3.1 (The R Foundation). Plots were made using RStudio (http://shiny.chemgrid.org/boxplotr/). In the figures, data points are plotted as shaded circles, centre lines show the medians, box limits indicate the 25th and 75th percentiles, and whiskers extend 1.5 times the interquartile range from the 25th and 75th percentiles.

Linear modelling was used to explore the effect of age at first vaccination (<8 vs 8–12 vs 12–16 weeks), sex, time point, individual vaccine group (groups 1–9), site of vaccine administration (scruff [groups 7–9] vs left hindlimb [groups 4–6] vs tail [groups 1–3]), and vaccine type (Fel-O-Vax 5 [groups 3/6/9] vs Fel-O-Vax Lv-K [groups 1/4/7] vs Leucogen FeLV [groups 2/5/8]) on anti-SU antibody ELISA levels (NOD) after FeLV vaccination. Negative NOD values were converted to zero for statistical analysis and plotting. Before analysis, variables were assessed using the Shapiro–Wilk test for normality, and any variables not meeting the assumptions for normality were log_e_ transformed for analysis. Univariable analyses were conducted to determine individual significance; any variables with a *P* value *<*0.25 were considered for inclusion in the multivariable model. A backwards stepwise elimination approach was used to determine the final multivariable model for each outcome (FeLV-A and FeLV-B) in which all terms were significant. Post-hoc Tukey’s tests were utilised to obtain pairwise differences. Results from the final multivariate model are shown unless the univariate result was insignificant. Statistical significance was considered when *P* <0.05 and NOD standard error reported for significant results.

FeLV-A and FeLV-B antibody responses at T2 (1 month after the second dose of FeLV vaccine had been administered) and T12 (12 months after the second dose of FeLV vaccine had been administered) were assessed separately to determine if an association was present with vaccine type/location and sex.

Vaccine ‘non-responders’ were classified as cats with a negative NOD against FeLV-A and/or FeLV-B at T2 (ie, sample OD ⩽negative control OD, for either FeLV-A/FeLV-B or for both). The prevalence of vaccine ‘non-responders’ based on vaccine type and site of vaccination was analysed using χ^2^ tests.

### Animal ethics

Animal ethics approval was obtained from the University of Sydney Animal Ethics Committee (approval number 2019/1165).

## Results

### Study population: kittens (n = 105)

In total, 125 kittens were recruited for the study, 10 animals fewer than planned. This was attributed to an unexpected FeLV vaccine shortage in Australia; Fel-O-Vax Lv-K and Leucogen FeLV were both discontinued by the manufacturers towards the end of the study period. This discontinuation was unrelated to the COVID-19 pandemic.

A further 20 kittens were lost from the cohort for the following reasons: seven due to the incorrect vaccine schedule being followed, seven due to owners withdrawing their animal from the study and six due to illness (unrelated to vaccination).

A total of 105 kittens, therefore, remained for post-vaccination analysis (Table 3). This cohort comprised 57 female and 48 male kittens, with a median age at recruitment of 13 weeks (range 7–16, interquartile range 9–14). Table 4 provides a summary of signalment by group. Three animals from the same litter (total litter size = 3) tested FIV-positive at all three kitten time points and were included in the analysis (kittens 97, 98 and 99 from groups 7, 8 and 3, respectively).

**Table 3:** Recruitment numbers for post-vaccination feline leukaemia virus (FeLV) antibody testing in kittens (n = 105, after exclusions)

Vaccination site	Fel-O-Vax Lv-K* (n = 42, 29)	Leucogen FeLV* (n = 30, 22)	Fel-O-Vax 5 (n = 33, 24)
Tail (n = 36, 26)	14 (9), group 1	10 (9), group 2	12 (8), group 3
Left hindlimb (n = 33, 25)	14 (11), group 4	9 (5), group 5	10 (9), group 6
Scruff (n = 36, 24)	14 (9), group 7	11 (8), group 8	11 (7), group 9

Adult cats that returned for re-vaccination and a fourth sample collection at day 365 are shown in brackets (n = 75, after exclusions). See ‘Study population: kittens (n = 105)’ and ‘Study population: adult cats (n = 75)’ sections for a complete list of exclusions

* Animals in these groups were concurrently administered Feligen 3 in the scruff, a modified-live virus core vaccine containing feline panleukopenia virus, feline herpesvirus-1 and feline calicivirus antigens, in addition to the FeLV vaccine administered into one of three different anatomical areas. Animals in groups 7 and 8, therefore, received two separate injections into the scruff

**Table 4:** Signalment details for each kitten group after exclusions (n = 105)*

Group	Median age at first vaccination (weeks)	Age range at first vaccination (weeks)	Females (n = 57, 54%)	Males (n = 48, 46%)
1	12	7–16	8 (57)	6 (43)
2	14	7–16	4 (40)	6 (60)
3	12	7–16	8 (67)	4 (33)
4	12	8–16	6 (43)	8 (57)
5	13	8–14	5 (56)	4 (44)
6	14	8–16	6 (60)	4 (40)
7	13	8–15	8 (57)	6 (43)
8	13	8–15	7 (64)	4 (36)
9	12	8–14	5 (45)	6 (55)

Data are n (%) unless otherwise indicated

* Kittens were enrolled into groups 1–9 consecutively, in order of presentation. All kittens were of mixed breed and were neutered before rehoming between day 0 (T0) and day 56 (T2)

Of the 105 kittens originally included, a further 22 were lost to follow-up, leaving 83 cats returning on day 365 (T12) for re-vaccination and a fourth blood draw.

### Study population: adult cats (n = 75)

Eight adult cats were excluded from the final analysis because an incorrect FeLV vaccine protocol had been followed on their final kitten visit (day 56). Therefore, 75 adult cats remained for analysis (40 females, 35 males) (Table 3). The three FIV-positive animals did not return for T12 sampling.

### Testing to determine FeLV infection and/or exposure

No progressive or regressive FeLV infections were detected in the entire kitten cohort (0/125, 0%).

A total of 14/125 (11%) kittens from seven different study groups (all except groups 2 and 5) and nine different litters tested FeLV NAb-positive at one or more of the three time points (eight females and six males; median age when testing positive = 17 weeks). Only 2/14 kittens tested NAb-positive at T0. Of the 14 kittens, 11 tested NAb-positive at a single time point (0 at the first time point, 3 at the second time point, 8 at the third time point), while 3/14 kittens tested NAb-positive at two time points (two kittens at T0 and T2, one kitten at T1 and T2). All NAb results were low titres (4 or 8) considered to be weak positives (Table 5). Two of the three FIV-positive kittens (kittens 97 and 98) tested NAb-positive (T2 only). No association was found between any specific vaccine and NAb positivity in kittens (*P* = 0.39; χ^2^ test).

**Table 5:** A total of 14 kittens from seven different study groups and nine different litters, and one additional adult cat from group 1 (cat 20), tested feline leukaemia virus (FeLV) neutralising antibody (NAb)-positive at various time points*

Group	Kitten/Cat	FeLV NAb result			
		T0	T1	T2	T12
1	92^a^ 20	0 0	0 0	8 0	No sample <8^†^
3	91^b^ 114	0 0	4 4	0 0	No sample 0
4	94^a^ 110^c^	0 4	0 0	8 8	No sample 0
6	101 116	0 0	8 0	4 8	0 0
7	89^b^ 97^d^ 112^c^	0 0 0	0 0 4	4 8 0	16 No sample No sample
8	98^d^ 106	0 0	0 0	8 8	No sample 0
9	88 111^c^	0 8	0 0	8 4	No sample 0

* Only groups 2 and 5 did not have kittens with NAb-positive results. A NAb titre of 4 was considered weakly NAb-positive and a titre of 32 was considered strongly NAb-positive. See Table 1 for group details and the ‘Vaccination schedule and vaccines administered’ section for vaccine formulation and manufacturer details. Kittens 97 and 98 were also feline immunodeficiency virus-positive

† This sample was extremely haemolysed and the 1:4 dilution was unreadable and therefore not able to be reported

a–d Kittens from the same litters

T0 = day 0; T1 = day 28; T2 = day 56; T12 = days 365

No progressive or regressive FeLV infections were detected in the entire cohort of adult cats at T12 (0/83, 0%).

Of the 14 previously NAb-positive kittens, seven presented at T12, with only 1/7 cats testing NAb-positive for FeLV at this time point, which was a cat in group 7 with a titre of 16 (cat 89). Another cat from group 1 was NAb-negative at all kitten time points but NAb-positive at T12 with a low titre (<8; cat 20). Hence, a total of 2/83 (2%) cats tested NAb-positive at T12 (Table 5).

### Serology testing for evaluation of FeLV-A antibody response after vaccination

A summary of the statistical analyses for FeLV-A antibody results is presented in Table 6.

**Table 6:** Results from univariate and multivariate analyses for feline leukaemia virus (FeLV-A) and FeLV-B antibody levels after vaccination*

Variable	Univariate analysis (*P* values)		Multivariate analysis (*P* values)	
	FeLV-A	FeLV-B	FeLV-A	FeLV-B
Age at first vaccination	0.45	0.26	−	−
Sex	0.001	0.005	0.003	0.009
Time (ie, boosting schedule)	0.015	<0.001	0.004	<0.001
Individual vaccine group	0.034	0.23	0.027	−
Site of vaccination	0.10	0.18	−	−
Vaccine type	0.42	0.079	−	−

* Variables were considered for inclusion in a multivariate analysis when the result from univariate analysis was *P <*0.25. Statistical significance from multivariate analysis was considered when *P* <0.05

#### Effect of age at first vaccination on FeLV-A antibody levels after vaccination

No effect of age at first vaccination (<8 vs 8–12 vs 12–16 weeks) on FeLV-A levels was observed (*P* = 0.45).

#### Effect of sex on FeLV-A antibody levels after vaccination

Female cats had significantly higher (1.65 times higher) FeLV-A antibody levels compared with male cats after adjusting for the effects of group and time (NOD 0.066 ± 0.007 vs 0.040 ± 0.005; *P =* 0.003). Pre-vaccination (T0) weight was not a confounding factor when factored into multivariable analysis of the FeLV-A antibody response by sex (ie, females did not receive a significantly higher vaccine antigen dose/kg than males; *P* = 0.90).

### Effect of boosting schedule on FeLV-A antibody levels after vaccination

**Figure 2:**
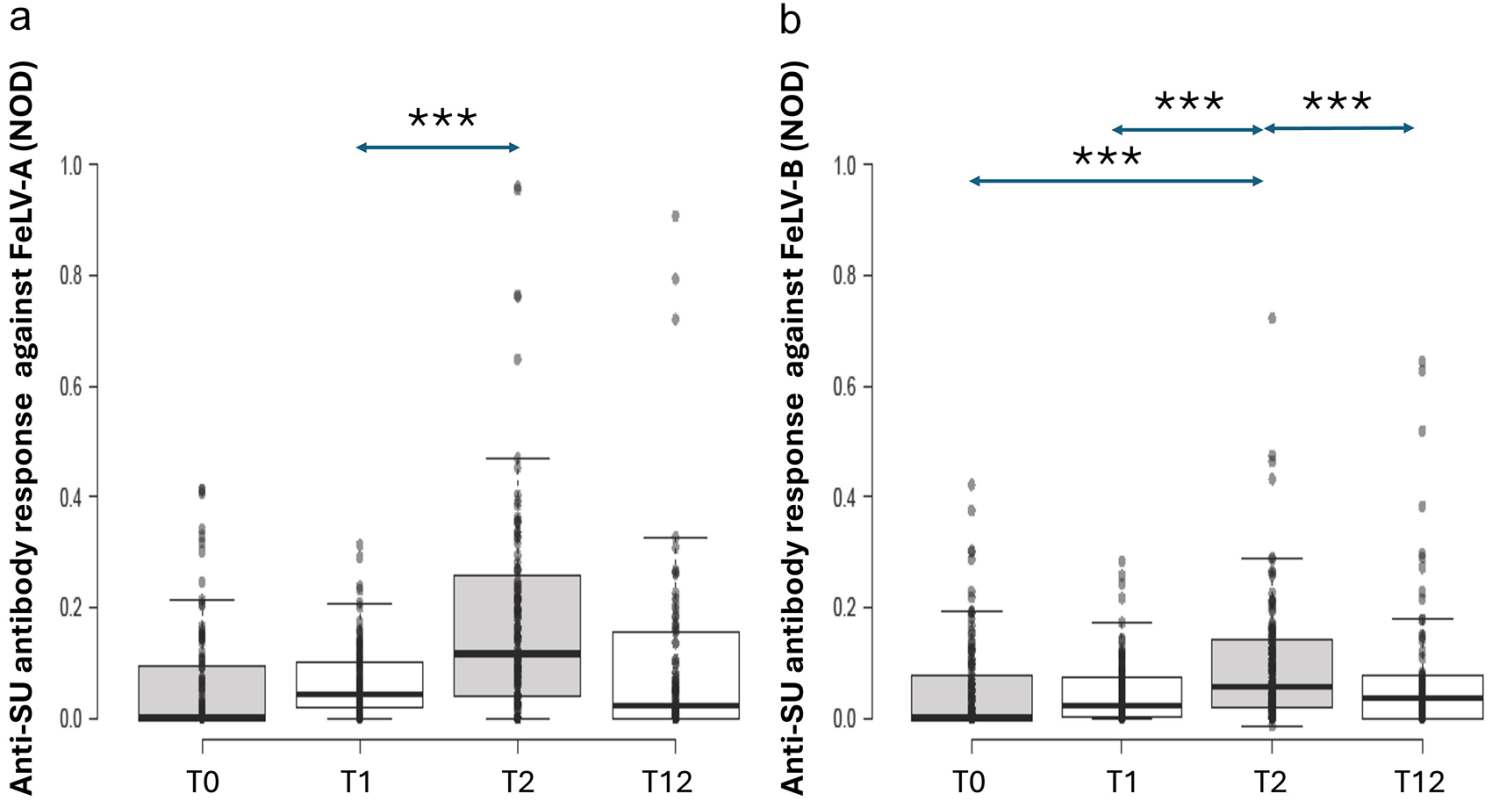
Original results of anti-surface unit (SU) antibody ELISA testing against (a) feline leukaemia virus (FeLV)-A and (b) FeLV-B at the four time points. T0 = day 0 (pre-vaccination), T1 = day 28 (1 month after first FeLV vaccination), T2 = day 56 (1 month after second FeLV vaccination), T12 = day 365 (1 year after second FeLV vaccination). With multivariable analysis, FeLV-A antibody levels at T2 were highest and were significantly higher than at T1 (*P* = 0.004), while FeLV-B antibody levels at T2 were significantly higher than all other time points (*P* <0.001). NOD = normalised optical density; *P*-value <0.01***

Overall, FeLV-A antibody levels at day 56 (T2) were highest and were significantly higher than at day 28 (T1) after adjusting for the other terms in the model (NOD 0.068 ± 0.010 vs 0.032 ± 0.005; *P =* 0.004) (Figure 2a).

At T2, 16 kittens had no measurable antibody response to FeLV-A (ie, vaccine ‘non-responders’).

#### Effect of individual vaccine group on FeLV-A antibody levels post-vaccination

Fel-O-Vax 5 administered into the tail (group 3) had significantly lower FeLV-A antibody levels compared withLeucogen FeLV administered into the tail (group 2), Fel-O-Vax Lv-K administered into the left hindlimb (group 4) and Fel-O-Vax 5 administered into the left hindlimb (group 6) (*P* = 0.027). All other individual group pairwise comparisons were not significantly different.

#### Effect of site of FeLV vaccine administration on FeLV-A antibody levels post-vaccination

Site of vaccine administration had no significant effect on overall FeLV-A antibody levels (*P* = 0.10).

**Figure 3:**
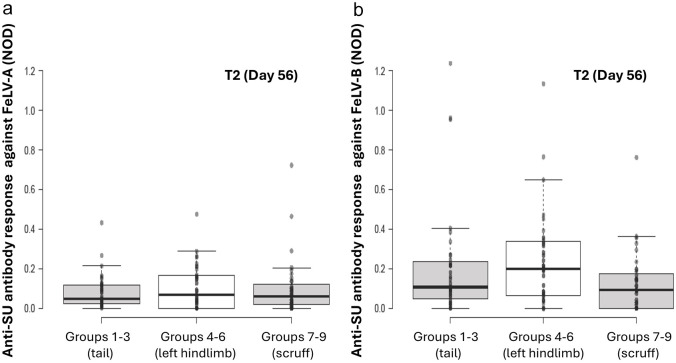
Original results of anti-SU antibody ELISA testing against FeLV-A (a) and FeLV-B (b) at time point 2 (T2; day 56) by site of vaccination, one month after the second dose of FeLV vaccine was administered. With univariable analysis, no significant differences between different sites of vaccine administration were observed FeLV = feline leukaemia virus; NOD = normalised optical density; SU = surface unit

**Figure 4:**
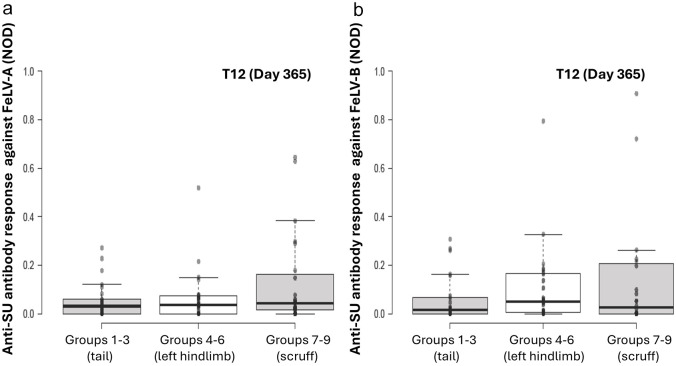
Original results of anti-SU antibody ELISA testing against FeLV-A (a) and FeLV-B (b) at T12 (day 365) by site of vaccination, one year after a primary course of FeLV vaccination was administered. With univariable analysis, no significant differences between different sites of vaccine administration were observed FeLV = feline leukaemia virus; NOD = normalised optical density; SU = surface unit

For time points T2 and T12 considered separately, there was no significant effect of site of vaccine administration on FeLV-A antibody levels (T2: *P* = 0.063; T12: *P =* 0.74; Figures 3a and 4a).

Tail vaccination produced significantly fewer ‘non-responders’ against FeLV-A at T2 than scruff and hindlimb vaccination (*P* = 0.020; χ^2^ test).

#### Effect of FeLV vaccine type on FeLV-A antibody levels post-vaccination

Vaccine type had no significant effect on overall FeLV-A antibody levels (*P* = 0.42).

**Figure 5:**
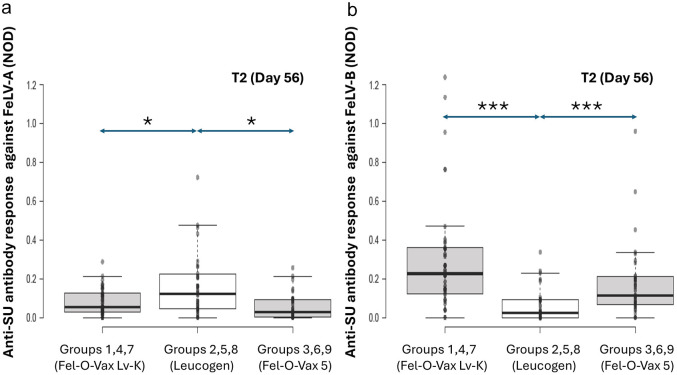
Original results of anti-surface unit (SU) antibody ELISA testing against feline leukaemia virus (FeLV)-A (A) and FeLV-B (B) at time point 2 (T2; day 56) by vaccine type (ie, Fel-O-Vax Lv-K vs Leucogen FeLV vs Fel-O-Vax 5), 1 month after the second dose of FeLV vaccine was administered. With univariable analysis, cats administered Leucogen FeLV produced higher FeLV-A antibody levels, but lower FeLV-B antibody levels, than the other two vaccines (*P* = 0.034 and *P* <0.001, respectively). NOD = normalised optical density; *P*-value <0.05 * and <0.01 ***

**Figure 6:**
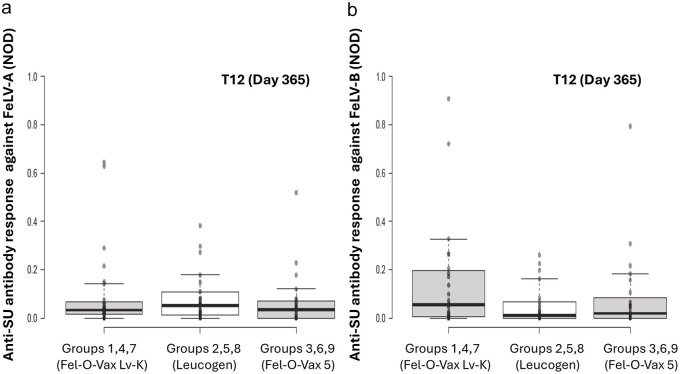
Original results of anti-surface unit (SU) antibody ELISA testing against feline leukaemia virus (FeLV)-A (A) and FeLV-B (B) at time point 12 (T12; day 365) by vaccine type (ie, Fel-O-Vax Lv-K vs Leucogen FeLV vs Fel-O-Vax 5), 1 year after a primary course of FeLV vaccination was administered. With univariable analysis, no significant differences between different vaccines were observed. NOD = normalised optical density

When time points T2 and T12 were considered separately, cats administered Leucogen FeLV produced significantly higher FeLV-A antibody levels irrespective of site of vaccination than the other two vaccines at T2 only (T2: *P* = 0.034; T12: *P =* 0.80; Figures 5a and 6a).

### Serology testing for evaluation of FeLV-B antibody response post-vaccination

A summary of the statistical analyses for FeLV-B antibody results is presented in Table 6.

#### Effect of age at first vaccination on FeLV-B antibody levels post-vaccination

No effect of age at first vaccination (<8 vs 8–12 vs 12–16 weeks) on FeLV-B levels was observed (*P* = 0.26).

### Effect of sex on FeLV-B antibody levels post-vaccination

Females had significantly higher (1.60 times higher) FeLV-B antibody levels compared with males after adjusting for the effect of time (NOD 0.083 ± 0.010 vs 0.052 ± 0.007; *P* = 0.009). Pre-vaccination weight was not a confounding factor when included in multivariable analysis of the FeLV-B antibody response by sex (*P* = 0.10).

#### Effect of boosting schedule on FeLV-B antibody levels post-vaccination

Overall, FeLV-B antibody levels at day 56 (T2) were significantly higher than all other time points after adjusting for the effect of sex (NOD 0.142 ± 0.024 [T2] vs 0.060 ± 0.013 [T0], 0.046 ± 0.008 [T1], and 0.047 ± 0.008 [T12]; *P* <0.001; Figure 2B).

At T2, 16 kittens had no measurable antibody response to FeLV-B (ie, vaccine ‘non-responders’). This included six kittens that had no measurable antibody response to FeLV-A or FeLV-B.

#### Effect of individual vaccine group on FeLV-B antibody levels post-vaccination

Individual vaccine group had no significant effect on overall FeLV-B antibody levels (*P* = 0.23).

#### Effect of site of FeLV vaccine administration on FeLV-B antibody levels post-vaccination

Site of vaccine administration had no significant effect on overall FeLV-B antibody levels (*P* = 0.18).

When time points T2 and T12 were considered in isolation, no significant effect from site of vaccine administration on FeLV-B antibody levels was observed (T2: *P* = 0.08; T12: *P =* 0.82; Figures 3b and 4b).

Scruff vaccination produced significantly more ‘non-responders’ against FeLV-B at T2 than tail and hindlimb vaccination (*P* = 0.032; χ^2^ test).

#### Effect of FeLV vaccine type on FeLV-B antibody levels post-vaccination

Vaccine type had no significant effect on overall FeLV-B antibody levels (*P* = 0.079).

For time points T2 and T12 considered separately, cats administered Leucogen FeLV produced significantly lower FeLV-B antibody levels irrespective of site of vaccination than the other two vaccines at T2 only (T2: *P* <0.001; T12: *P =* 0.43; Figures 5b and 6b).

Leucogen FeLV vaccination produced significantly more FeLV-B ‘non-responders’ at T2 than the other two vaccines (*P* = 0.0095; χ^2^ test).

## Discussion

Overall, animals vaccinated with different FeLV vaccines (Fel-O-Vax 5, Fel-O-Vax Lv-K and Leucogen FeLV), irrespective of the anatomical site of injection (scruff, left hindlimb or tail), did not demonstrate any biologically significant differences in FeLV antibody levels. The lack of meaningful differences in antibody response between vaccine types or administration sites may help veterinary advisory bodies and clinicians make informed, evidence-based recommendations regarding FeLV vaccination sites.

Both FeLV-A and FeLV-B antibody levels increased in kittens after administration of a second vaccine in the primary course, as expected, due to the so-called anamnestic response (‘booster’ effect) that develops after initial exposure to an antigen and priming of the immune system. However, the antibody response between individuals to a course of vaccination was inconsistent, with a wide and highly variable serological response observed, likely due in part to individual animal factors. A proportion of individuals developed high antibody concentrations while, somewhat unexpectedly, a substantial number of cats had a much smaller response or even no antibody response at all to vaccination. The value of FeLV re-vaccination 1 year after a primary vaccination course was indicated by the significant reduction in FeLV-B antibody values over the period from T2 to T12; FeLV-A antibody levels also decreased over the same period, although the decrease was not statistically significant.

Positive and negative cut-off values for the FeLV anti-SU ELISA used have not yet been established, and protective titres remain undefined. This made assessing the significance of any antibody concentration differences between vaccine groups difficult. When the anti-SU ELISA was first reported, a significant difference in mean antibody responses was noted between cats with progressive and regressive infections; however, a wide range of results and substantial overlap between infection categories limited its discriminatory ability.^48^ In the present study, with univariable analysis, cats administered Leucogen FeLV produced higher FeLV-A antibody levels, but lower FeLV-B antibody levels, than the other two vaccines at T2. Vaccination with Leucogen FeLV also produced more ‘non-responders’ against FeLV-B at T2 than Fel-O-Vax 5 and Fel-O-Vax Lv-K. This difference between vaccines was difficult to explain, since all three vaccine formulations contained FeLV-A antigen and none contained FeLV-B antigen. Fel-O-Vax 5 administered into the tail produced significantly lower FeLV-A antibody levels compared with several other groups, including Fel-O-Vax 5 administered into the left hindlimb; however, tail vaccination produced significantly fewer ‘non-responders’ against FeLV-A than scruff and hindlimb vaccination. Considering these confusing and sometimes conflicting results, we believe any differences in post-vaccinal antibody levels found with vaccine type or site of vaccination were small and unlikely to be biologically significant.

In a previous study, FeLV-naive cats vaccinated in the right flank fold with monovalent Fel-O-Vax Lv-K exhibited a higher median antibody response than FeLV-naive cats vaccinated in the scruff with Fel-O-Vax 5, using the same anti-SU ELISA used in the current study.^33^ Fel-O-Vax Lv-K and Fel-O-Vax 5 contain the same IWV antigen from a single subtype isolate (FeLV-A/61E), produced by the same manufacturer in the same facility. Consequently, this observed difference in antibody response was unexpected. As this previous work was a retrospective study, and cats had been administered vaccines in different anatomical locations, it was not possible to ascertain what might have been the cause of the difference. We had several hypotheses, including site of vaccine administration and differences in lymphatic drainage, an adjuvant effect (approximately two times the volume of adjuvant was administered with Fel-O-Vax Lv-K compared with Fel-O-Vax 5 because of concurrent vaccination with Fel-O-Vax 3), a ‘batch’ effect associated with variation in antigenic potency values between batches of Fel-O-Vax Lv-K and Fel-O-Vax 5 used, and antigenic competition with the polyvalent Fel-O-Vax 5 vaccine (although this phenomenon is more likely to occur with immunologically related vaccine antigens).^33^ Administration of vaccines into the right flank fold was not considered in the present study, as there are no major vaccination bodies that advise vaccinating into the flank as an alternative to the scruff.^16,17^

Several studies about human medicine have found that the anatomical injection site can affect vaccine immunogenicity. For most vaccines, the recommendation is that they are administered intramuscularly (rather than subcutaneously), typically into the deltoid or the anterolateral aspect of the thigh to optimise the immunogenicity of the vaccine and to minimise adverse reactions at the injection site.^49–51^ Administration of a hepatitis B vaccine into the upper arm produced an antibody titre up to 17 times greater than when administered into the buttock.^52^ Administration of a booster messenger ribonucleic acid (mRNA) COVID-19 vaccine into the contralateral deltoid muscle (relative to initial vaccination) resulted in significantly higher serum IgG levels than a booster vaccination into the ipsilateral arm.^34^ Recently, however, contradictory results from another study using the same mRNA COVID-19 vaccine were reported, with people who received both doses in the same arm producing significantly faster antibodies within the first week after the second dose than when administered in alternating arms.^35^ In the current study, we speculated that tail vaccination likely resulted in some intramuscular injection due to negligible subcutaneous space. In comparison, a correctly administered scruff or hindlimb vaccination would be solely subcutaneous. We questioned whether this difference could have contributed to the lower number of FeLV-A ‘non-responders’ in cats vaccinated in the tail. A further theory would be that there is likely variation in blood supply across different tissue types, which could affect the accessibility of immune cells, such as macrophages and dendritic cells, to the tissues where the vaccine is administered, and these cells are essential for antigen recognition, processing and presentation to lymphocytes.^53,54^ Proximity to lymphatic drainage may also play a role.^55^ The hypothesis that there may be differences in immune response after vaccination between different anatomical locations in the feline patient, therefore, seems reasonable and warrants further investigation.

A total of 15 different animals (14 kittens and one adult cat) tested FeLV NAb-positive in the study. NAbs to FeLV are believed to develop in vaccinated cats after experimental or natural exposure, although results from some studies have suggested that NAb production in FeLV-vaccinated cats before challenge or without natural exposure is possible.^14,56,57^ Although no association was found between any specific vaccine type and NAb positivity in kittens, the results from this study could support this theory. The possibility of NAb-positive results in kittens from MDAs was also considered; however, given that 12/14 kittens tested NAb-negative at T0, this seemed unlikely. Instead, we propose that the most probable explanation for the NAb-positivity rate in kittens (14/125, 11%), despite owners being advised to confine their kittens completely indoors until the completion of the primary vaccination series, was natural FeLV exposure during their primary vaccination course. A survey of cat owners in Australia and New Zealand indicated that while 40% of owners had intended to house their cat completely indoors when first acquired, 83% of cats ended up having some level of access to the outdoors.^58^ As a result of the field nature of the study (as opposed to SPF kittens housed inside a laboratory), natural exposure to FeLV during the study period was possible. A previous study in Australia reported a similar prevalence of abortive infections in 11% of pet cats with outdoor access.^14^

The most unexpected and novel finding from this study was the strong sex-linked difference in serological response to vaccination, with a higher antibody response against both FeLV-A and FeLV-B observed after vaccination in female cats compared with male cats. This has been a general trend in all mammalian species, including adult humans vaccinated with both live and inactivated vaccines (eg, influenza, hepatitis B, yellow fever, rabies, genital herpes and smallpox).^59^ There have been few robust investigations of the possible effect of sex on vaccination response in cats to date. In a study of rabies vaccination responses in cats, the authors observed a marked sex effect, with entire males least likely to develop a protective response to vaccination. That study also highlighted the important role of sex hormones in antibody response, with entire animals (both males and females) having a greater risk of an inadequate antibody response compared with neutered animals.^60^ This finding is particularly relevant given that all kittens in the present study were neutered between T0 and T2, before reaching sexual maturity and the associated increase in production of sex steroid hormones. Gonadal steroid hormones (eg, oestradiol, progesterone and testosterone) play an important role in modulating the immune response, including suppressing B cell numbers (depending on the antigen involved). Furthermore, castration of male rats has been shown to induce a general increase in antibody levels, as well as cell-mediated immunity (CMI) responses.^61^ Other factors apart from sex, such as age, breed, nutrition and overall health status, can also have an impact on vaccine response in cats.^16^ Further work in this area to advance our understanding is warranted.

It was not possible to determine whether the sex difference observed translates to a difference in risk of progressive infection after natural challenge. The protective levels of FeLV-A and FeLV-B anti-SU ELISA antibodies to define an effective humoral immune response have not been defined. Furthermore, protection against FeLV infection involves both humoral immunity and CMI; therefore, measuring antibodies alone might not be sufficient to determine protection against FeLV challenge.^44,62,63^ Indeed, some studies have suggested CMI could play a more important role than humoral immunity after FeLV vaccination.^3,43,44,64,65^ It is possible that animals might still be afforded protection from FeLV challenge, despite a low antibody response after vaccination (eg, the vaccine ‘non-responders’ in the current study). In the six combined FeLV-A and FeLV-B ‘non-responder’ kittens, no sex bias was noted (three female, three male). Across a diverse range of species, including humans, males are more susceptible than females to many viral infections, as females tend to mount both stronger innate and adaptive immune responses.^66^ A pertinent veterinary example is feline infectious peritonitis.^67–69^ Although this often results in more efficient clearance of pathogens, and greater vaccine efficacy is observed in females compared with males, the tradeoff is that there is also an association in females with increased susceptibility to the development of inflammatory and autoimmune diseases.^70^ There is potential for future investigations to assess the magnitude of the CMI response to FeLV vaccination (eg, cytotoxic T lymphocyte levels), including potential differences between FeLV vaccine types and sites of vaccine administration, and exploring differing responses to FeLV challenge between males and females.

In epidemiological field studies, male cats have often been reported to have a higher risk of progressive FeLV infection compared with females, presumably due to increased at-risk behaviours (ie, fighting).^6,71–76^ When investigating two previous FeLV outbreaks, which occurred within group-housed cohorts in Australia, we observed no sex bias in the development of either progressive or regressive infections.^14^ To the authors’ knowledge, no predilection to FeLV infection in males compared with females has ever been reported in experimental FeLV challenge studies, although study group sizes were often too small to detect such an effect. It seems plausible, given the findings from the current study, that males might be more at risk of progressive FeLV infection than females because of a reduced ability to mount an effective humoral immune response after challenge. In this scenario, males would be less able to clear localised infection of the oropharyngeal tissue before the virus spreads systemically.^77^ Future studies could explore this hypothesis.

Although this study demonstrated a comparable antibody response to vaccination regardless of FeLV vaccine type or administration site, these findings cannot be extrapolated to indicate equivalent protection against FeLV challenge. Fel-O-Vax 5, Fel-O-Vax Lv-K and Leucogen FeLV have reported efficacies in the range of 44–100%, 86–100% and 68–87% respectively, based on experimental challenge studies.^36,43,44,47,57,78–80^ However, it must be noted that sample sizes were often small and therefore studies were likely underpowered. To date, only laboratory-based studies, rather than field-based studies, have been performed to evaluate FeLV vaccine efficacy.^33^ One would think that in highly endemic countries, such as Thailand or Brazil, field studies of FeLV vaccine efficacy would be readily performed and prove insightful.^81–84^

Critically, there is a general lack of pharmacovigilance studies to monitor suspected vaccine ‘breakthrough’ infections and evaluate the occurrence of such events in the field. Given the high prevalence of abortive FeLV cases previously reported in Australia, and those suspected in the current study, we strongly recommend FeLV vaccination of at-risk populations of cats in this country with one of these three FeLV vaccines, unless cats are destined to live in an exclusively indoor environment, free of FeLV-infected cats.^14^ In particular, FeLV vaccination should be considered in young kittens and cats aged less than 1 year with unsupervised outdoor access because of an age-related susceptibility to infection.^85–87^ These recommendations are consistent with current vaccination guidelines published by AAHA and the AAFP, and the World Small Animal Veterinary Association.^16,17^ It is unfortunate that no monovalent FeLV vaccines are currently available in Australia. This absence makes it difficult to implement the aforementioned recommendation, as the only available option involves using a heavily adjuvanted polyvalent inactivated vaccine.

## Conclusions

Vaccination of cats at the subcutaneous scruff, left hindlimb or tail using different FeLV vaccine formulations generally elicited comparable humoral immune responses. This suggests that alternative sites to the scruff can be considered effective and are justified options for FeLV vaccination. However, further research is needed to better understand the immune cell populations involved in generating immunity at these different anatomical sites after vaccination. Future studies will also investigate the influence of sex on vaccine-induced immunity and explore whether a sex-dependent predisposition to FeLV infection exists. Given the potential exposure risks (11% in kittens and 2% in adults), Australian veterinarians should consider the need for FeLV vaccination in at-risk kittens and young adult cats.
